# The First Record of Whitefly (Hemiptera, Sternorrhyncha, Aleyrodidae) from Bitterfeld Amber

**DOI:** 10.3390/insects17010050

**Published:** 2025-12-30

**Authors:** Jowita Drohojowska, Anita Gorzelańczyk, Natalia Tomanek, Małgorzata Kalandyk-Kołodziejczyk, Jacek Szwedo

**Affiliations:** 1 Institute of Biology, Biotechnology and Environmental Protection, Faculty of Natural Sciences, University of Silesia, 9, Bankowa St., PL 40-007 Katowice, Poland; jowita.drohojowska@us.edu.pl (J.D.); anita.gorzelanczyk@us.edu.pl (A.G.); n_tomanek@tlen.pl (N.T.); malgorzata.kalandyk@us.edu.pl (M.K.-K.); 2 Laboratory of Evolutionary Entomology and Museum of Amber Inclusions, Department of Invertebrate Zoology and Parasitology, University of Gdańsk, 59, Wita Stwosza St., PL 80-308 Gdańsk, Poland

**Keywords:** Insecta, whiteflies, Aleyrodinae, fossil, Eocene

## Abstract

A male specimen of whitefly *Pudrica christianottoi* Drohojowska et Szwedo, 2024, of subfamily Aleyrodinae, previously known from the sole female specimen from Lower Lusatia succinite, is here described, based on an inclusion from Bitterfeld amber. This fossil is contributing new data to our understanding of morphological disparity, sexual dimorphism, taxonomic diversity and palaeobiogeographic distribution of the whiteflies in the Eocene fossil resins. It is also a contribution to the ongoing discussions on age, similarities, dissimilarities and taphonomic differences among Eocene resins of Europe collectively known as ‘Baltic amber’.

## Simple Summary

Scientists have found a male whitefly fossil in Bitterfeld amber. It is a species called *Pudrica christianottoi* Drohojowska et Szwedo, 2024. Until now, this species has only been found as a single female from Lower Lusatia amber. The fossil species is a member of the family Aleyrodidae of the insect order Hemiptera. These small, inconspicuous insects live on and feed off plants, often being unnoticed as they dwell on the underside of leaves. There are more than 1700 species of whitefly worldwide. These insects are mainly sorted into groups based on their larval form, called the puparium. The fossil record of whiteflies includes over 30 species, found as mostly as adults, and exceptionally as puparia. The new specimen provides new data about the imago’s morphology and shows how sexual dimorphism creates differences in appearance. The fossil male was found in a different area, so it also gives us new information about where the whiteflies that are now extinct were found. It also adds to the ongoing discussion about the age, similarities and differences in how Eocene resins in Europe are formed.

## 1. Introduction

It is a widely accepted view that insects represent the most diverse group of animals on Earth, in terms of both taxonomic diversity and ecological function. The Hemiptera is a notable component of the recognised taxonomic diversity and morphological heterogeneity, constituting the most substantial monophyletic order of the hemimetabolous insects and representing one of the five insect orders with the highest species diversity [1,2,3]. The order Hemiptera is further categorised into six sub-orders, namely the extinct Paleorrhyncha and extant Sternorrhyncha, Fulgoromorpha, Cicadomorpha, Coleorrhyncha and Heteroptera. It comprises over 330 families, known since the Late Carboniferous, with more than 100,000 extant species described [1,3,4,5,6,7]. The Sternorrhyncha, comprising 19,000 described extant species, are of significant ecological and economic importance [2,6,8]. The fossil record of these hemipterans extends back to the Early Permian; however, their evolutionary history has its origins in the Late Carboniferous, and the early stages of its evolution and diversification remain to be fully elucidated. The family Aleyrodidae Westwood, 1840, is commonly referred to as whiteflies, a denomination derived from the presence of a powdery secretion that covers the bodies and wings of the adult species. These insects are minute in size, with a body length and wingspan measuring less than 3 mm, and a body length measurement of 1–2 mm. They always feed on the undersides of leaves as nymphs, puparia and adults. The majority of species exhibit a high degree of relatedness with specific host plants [9,10,11]. Should numerical values of species be utilised as an accurate guide, whiteflies would be by far the least speciose of the four major extant infraorders of sternorrhynchans, with approximately 1700 currently valid extant species. The family Aleyrodidae is currently subdivided into four subfamilies: the extinct Bernaeinae Shcherbakov, 2000, the extant Aleyrodinae Westwood, 1840, the Aleurodicinae Quaintance et Baker, 1913, and the Udamoselinae Enderlein, 1909; the latter has been deemed to have questionable taxonomic status [11]. The fossil record of the family Aleyrodidae extends to the Late Jurassic [12,13]. Other fossils have been reported from the sedimentary deposits (although these records are very scarce) and are more commonly encountered as inclusions in fossil resins from the Lower Cretaceous to the Pliocene [11,14].

Here, we present the description of a male of *Pudrica christianottoi* Drohojowska et Szwedo, 2024, a species previously known from a single female found in succinite from the Miocene deposits of Lower Lusatia (Germany). This finding contributes to our understanding of the diversity and disparity of fossil whiteflies, and provides insights into depositional and taphonomic conditions, as well as the history of the fossil resins.

## 2. Materials and Methods

### 2.1. Geological Setting

The classification of fossil resins is determined by their chemical composition, geographical and botanical origins. The most common type of amber in Europe is succinite, a polymer of resins from gymnosperm trees, which contains 3–8% percentage of succinic acids [15,16,17]. Succinite is sourced from several locations, but the most prevalent kind is ‘Baltic amber’, originating from the Gulf of Gdańsk area. This fossil resin probably formed during the Eocene as a result of resinous exudations from the plant families Sciadopityaceae or Pinaceae [18], plants that probably formed “Baltic amber forests”, which are thought to have stretched along the margins of the North Sea during the Paleogene [19]. Succinite has also accumulated in the Rovno region (Ukraine), and it is believed that this material most likely originated from coniferous trees growing in the Ukrainian Crystalline Rock Massif, which was characterised by a subtropical climate [20]. A third substantial deposit of succinite has been identified in the vicinity of Bitterfeld (Saxony-Anhalt, Germany), sometimes referred to as Saxonian amber, with resins excavated from a decommissioned open-cast “Braunkohle” (brown coal) mine at Goitzsche.

A significant aspect that merits attention is the long-standing controversy surrounding the age of this amber and accessory resins (see, e.g., reviews in [21,22]). The provenance of resins has been linked to the Bitterfelder Bernsteinschluff Horizont [22,23,24], which comprises layers of Upper Oligocene age (Chattian, 23.0–28.1 Ma; [22,25,26]), but there is evidence that the resins deposited there may be older and were reworked into those horizons later [22,27]. It has been determined that these layers also contain accessory resins, with some of them being of angiosperm origin. Furthermore, the composition and amount of these resins differ from those found in deposits from the Gulf of Gdańsk area [17,22,28,29]. It has been argued that Bitterfeld and Baltic succinite are not synonymous and geochemically divergent [18,30,31]; on the other hand, their similarity or coeval age was suggested (but see discussions, e.g., in [21,32,33]. The debate on temporal conspecificity of the Baltic, Bitterfeld, Lusatian and other succinites from Central Europe is not entirely trivial, as it impacts questions regarding the potential longevity of plant and arthropod species, or their wider lineages, in essentially unchanged states throughout the Cenozoic era of northern Europe.

In this particular context, we identified the first whitefly from Bitterfeld amber, representing the same species as previously reported from amber from Lower Lusatia [14].

### 2.2. Morphology and Documentation

The specimen studied is a piece of Baltic amber (succinite), from the Bitterfeld deposit, and comes from the collection of Hans Werner and Christel Hoffeins (Germany). Observations were conducted at the Institute of Biology, Biotechnology and Environmental Protection, University of Silesia, employing Nikon SMZ25, Nikon SMZ1500, Nikon SMZ1270, and Leica M205C stereoscopic microscopes and a Nikon Eclipse E600 microscope; observations and documentation were made with direct and transmitted light (Nikon, Tokyo, Japan; Leica, Wetzlar, Germany). Additional observations and photographs were made with a Nikon Microphot-FX microscope and a Nikon DS-Fi2 digital camera with a DS-U3 controller, under the control of NIS Elements D 5.20.01 software, and with a Nikon Eclipse E600 microscope with a Nikon DSFi2 digital camera, operating under the control of NIS Elements D 4.20.03 software; and Nikon SMZ1500 with a Nikon DS Fi3 camera, under the control of NIS Elements D 5.01.00 software, the photographs were edited using Adobe Photoshop Elements 6.0. Photographs were also captured in the Laboratory of Evolutionary Entomology and Museum of Amber Inclusions, Department of Invertebrate Zoology and Parasitology, University of Gdańsk, using an Olympus SZX10 equipped with an EP50 digital camera (both Olympus Corp. Tokyo, Japan) and EPview Version 3.7.2 (Olympus Soft Imaging Solutions GmbH freeware, Münster, Germany). The photographs were readjusted using CorelDRAW X7 (Corel Corporation, Ottawa, Canada). Drawings were made with the aid of a camera lucida attached to an Olympus SZX10 stereomicroscope. The morphological terminology is given after Drohojowska And Szwedo [34].

## 3. Results

### Systematic Palaeontology

Order Hemiptera Linnaeus, 1758 [35].

Suborder Sternorrhyncha Amyot et Audinet-Serville, 1843 [36].

Infraorder Aleyrodomorpha Chou, 1963 [37].

Family Aleyrodidae Westwood, 1840 [38].

Subfamily Aleyrodinae Westwood, 1840 [38].

Genus *Pudrica* Drohojowska et Szwedo, 2024 [14].

Type species. *Pudrica christianottoi* Drohojowska et Szwedo, 2024; by original designation and monotypy.

**Figure 1:**
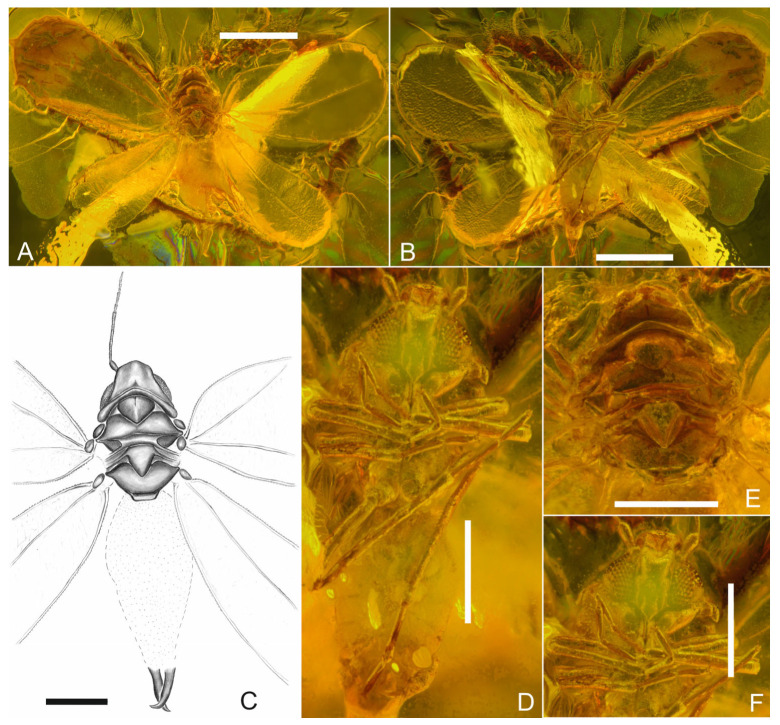
*Pudrica christianottoi*, male: (**A**,**C**) Body in dorsal view. (**B**,**D**) Body in ventral view. (**E**) Head and thorax in dorsal view. (**F**) Head and legs in ventral view; scale bars: 0.5 mm for (**A**,**B**); 0.2 mm for (**C**–**F**).

**Figure 2:**
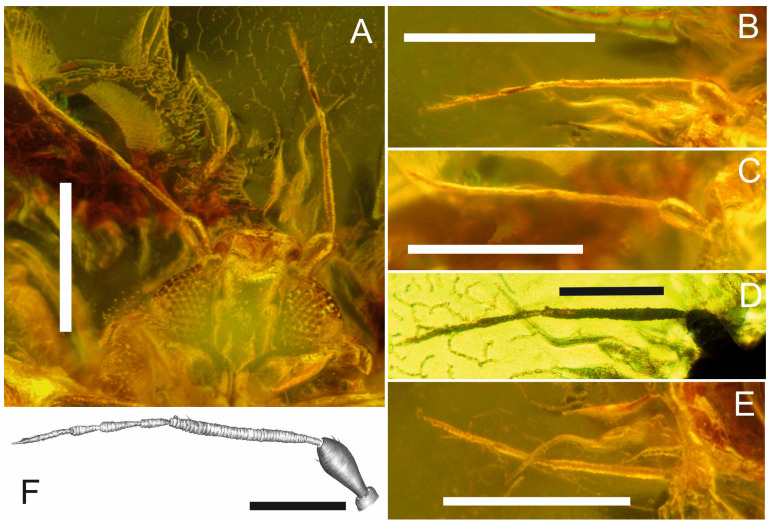
*Pudrica christianottoi*, male: (**A**) Head with antennae in ventral view. (**B**–**F**) Antenna; scale bars: 0.2 mm for (**A**–**C**,**E**); 0.1 mm for (**D**,**F**).

**Figure 3:**
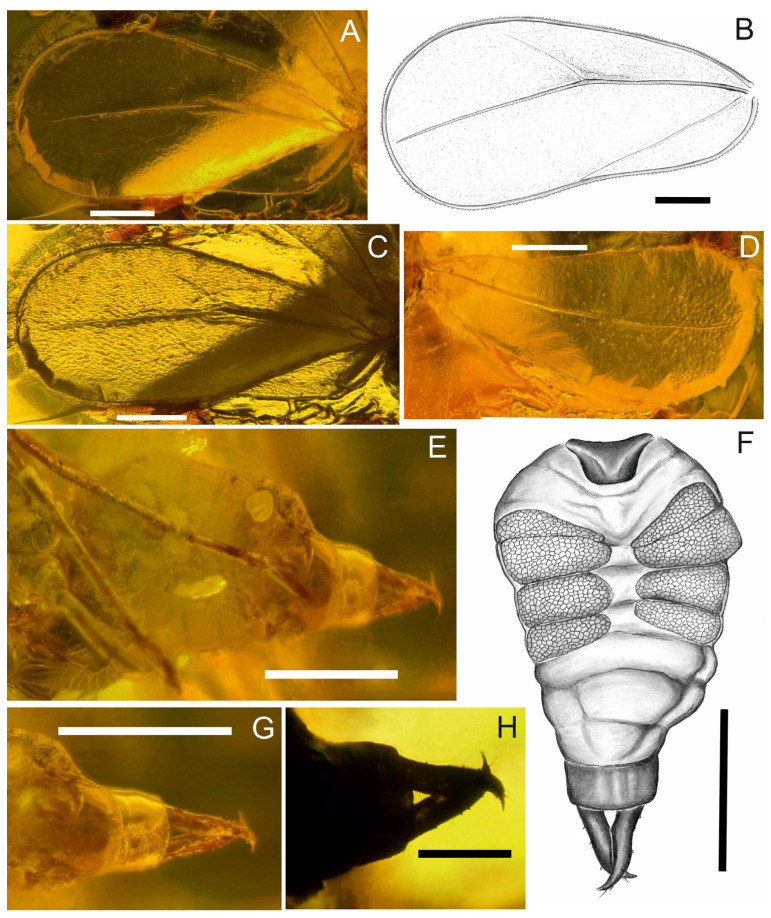
*Pudrica christianottoi*, male: (**A**–**C**) fore wing. (**D**) hind wing. (**E**,**F**) abdomen. (**G**,**H**) genitalia. Scale bars: 0.2 mm for (**A**–**G**); 0.1 mm for (**H**).

*Pudrica christianottoi* Drohojowska et Szwedo, 2024.

(Figure 1A–F, Figure 2A–F and Figure 3A–G).

*Emended diagnosis.* In general, features and venation pattern similar to *Snotra* Drohojowska et Szwedo, 2016, with antennae 7-segmented, with antennomeres F4 and F6 the same length (different length in *S. christelae*; in *S. herczeki* antennae 6-segmented); antennomeres F3 and F5 with subapical rhinaria; compound eyes divided, connected by four ommatidia (compound eyes not dived in *Snotra*); thorax with mesopostnotum slightly concave in median portion, lateral sections convexly declivous laterad; forewing less than 2.3 times as long as wide (as in *S. herczeki*; in *S. christelae* 2.5); vein Sc + R almost straight, not forked (Sc + R curved in apical half, not forked in *S. herczeki*; vein Sc + R bent in apical half, weakly forked into Sc + Rs in *S. christelae*); CuP (claval fold) distinct, with apex reaching half of fore wing length (not reaching half of fore wing length in *Snotra*).

*Description.* Body length approx. 1.03 mm. *Head* with compound eyes is 0.9 times as wide as the pronotum (Figure 1A,C,E), trapezoidal in shape from the dorsal side (Figure 1A,C,E) with an indistinct depression in the middle (traces of coronal suture perhaps); lateral margins merely sinuately diverging posteriad, posterior margin concave, disc of vertex concave; median ocellus absent, lateral ocelli distinct above the compound eyes, at level of anterior line of compound eye; compound eyes in dorsal view flattened, not bulging, divided, connected in median portion with 4 ommatidia, ommatidia of equal size. Rostrum (Figure 1D,F) short and massive, reaching the hips of the second pair of legs. Antennae seven-segmented (Figure 2A–F), scapus short, about ⅓ as long as pedicel, pedicel club-shaped, twice as long as wide, ca. 2.3 times shorter than the longest third segment, antennomeres F4 and F6 of same length, antennomere F7 longer than antennomere F6, with apical constriction (concavity) and single apical seta; subapical rhinaria visible on antennomeres F3 and F5; F3 with subapical seta, F7 tapers bilaterally towards the end; the cumulative length of F4, F5, F6 and F7 subequal to the length of F3.

*Thorax* (Figure 1A,C,E) is clearly visible. Pronotum 12 times wider than long in the midline, arcuate, anterior margin arcuate, diverging posteriad, lateral margins broader than the length of the pronotum in the midline, with lateral margins rounded, posterior margin deeply arcuate; mesopraescutum 1.37 times as long as broad, anterior margin arcuate, posteriad margins converging, straight, apex slightly rounded, with angle slightly wider than rectangular angle; mesoscutum slightly wider than pronotum, narrowed in the middle and 9 times wider than longer in the midline; anterior margin arcuate, lateral angles convex, with anterior lateral angles arcuate and posterior lateral angles less protruding and more pointed; lateral parts in the form of relatively long, curved cones, whose apices run towards the rear of the body; parapteron large, ellipsoid, flattened; mesoscutellum is a horizontal band with a slightly convex anterior margin and an arched posterior margin; posterior lateral parts in the form of elongated bands directed towards the rear of the body; mesopostnotum subtriangular, with median shallow concavity, lateral portions convexly declivous laterad; lateral bands of mesoscutellum and mesopostnotum visible; metascutum narrowed, 6 times wider than its length in the median line, where the narrowing occurs; anterior margin arched concave, anterior lateral and posterior lateral angles gently rounded; lateral parts in the form of short bands with apices directed towards the front of the body; metascutellum parts visible, broad, about 4 times as wide as long in midline, anterior and posterior margins straight.

*Legs* well visible; metatibia the longest, proleg appears the shortest; metacoxa well visible, cylindrical in shape; metatibia 1.3 times longer than the combined length of metatrochanter and metafemur; tarsi 2-segmented, basitarsomere longer than apical tarsomere; tarsal claws visible, slightly curved; paronychium not visible except on one of the metalegs as a short, relatively wide and blunt structure; basimetatarsomere 1.5 times longer than apical metatarsomere; all legs covered with bristles; pro- and metalegs (Figure 1B,D), short hairs are scattered on the tibia, and there are also single bristles on the tarsi; metalegs (Figure 1B,D) covered with bristles on the shins, where there is also a comb with at least a dozen long, relatively evenly spaced bristles; single, short hairs are also present on both tarsal segments.

*Fore wings* (Figure 1A,B and Figure 3AC) visible, left one, in the distal section covered with a milky-brown structure, right one bent at the wing joint so that the anterior and posterior sections of the wing are reversed; fore wing (Figure 1A,B and Figure 3A–C) widens towards the apex, reaching its widest dimension at approximately ⅔ of its length; it is about 2.1 times longer than wide; costal margin gently arched, convex; the anteroapical angle, apical margin and posteroapical angles broadly rounded; the posterior margin rounded at the base, with a slight indentation that begins before reaching the middle of the wing length; anterior, apical and posterior edges of the wing with a distinct, granular surface; common stem Sc + R thickened, well defined, running towards the anteroapical angle and branching just before reaching the middle of the wing; branch R1 slightly curved and relatively short; branch Rs, approximately 2.8 times longer than R1, straight, approaches almost to the fore wing apex; branch CuP runs in an almost straight line to the shallow concavity in the posterior margin of the fore wing, separating claval margin and tornus, but does not reach the margin.

*Hind wing* (Figure 1A,B and Figure 3D) slightly upturned, with the left one having an incomplete apical part; hind wing widened in the distal part, about 2.3 times longer than wide, reaching its maximum width at approximately ⅔ of its length; anterior margin straight, with a slight indentation at ⅓ of the length; anteroapical angle and apical margin arched, posteroapical angle broadly rounded, posterior margin almost straight; edges of the hind wing granular; common stem Sc + R thickened and clearly visible, smoothly transitioning into Rs, with a slightly arched course towards the apex, but not reaching the margin.

*Abdomen* (Figure 1A,B) longer than the combined length of head and thorax, invisible from the dorsal side except for the shape of the claspers; four wax fields visible from the ventral side (Figure 3E,F); male copulatory apparatus (Figure 3E–H) 1.6 times longer than wide at base; aedeagus not visible; claspers, constitute 69% of the length of the copulatory apparatus, cross at about ⅓ of their length, after which their sharp tips curved mediad and extended beyond the outer edge of the adjacent claspers; outer margin of the claspers straight, while the inner edge first bulges outwards and then curves inwards; claspers equipped with bristles, also in the distal part, in addition.

For measurements, see Table 1.

*Specimen.* Male. Hans Werner and Christel Hoffeins collection nr 834-1, deposited in the collection of the Museum of Amber Inclusions of the University of Gdańsk, MAIG 7290 Gdańsk, Poland. A flat box-shaped piece of amber (0.7 cm side length), small and thin, embedded in transparent resin with a large margin around it (according to [39]). The amber is light in colour, without any impurities. The specimen under examination is located in the centre of the lump in a dorso-ventral position and within a ‘sun spangle’, discoidal crack with numerous cracks.

*Locality and stratum*. Goitzsche Lagerstätte (former Goitzsche mine), Bitterfeld-Wolfen, Sachsen-Anhalt, Germany; Bitterfeld amber (Late Eocene in age, see Discussion for details) from the Cottbus Formation of Upper Oligocene: Chattian, 23.0–28.1 Ma [40,41].

## 4. Discussion

The specimen described above exhibits all distinguishing morphological characters of *Pudrica christianottoi* Drohojowska et Szwedo, 2024, but it is a male specimen. As demonstrated in Table 1, the male specimen is observed to be marginally smaller than the female. This feature has been documented as being prevalent among whiteflies. However, an analysis of the proportions of body parts and crucial elements revealed them to be identical, thus enabling identification of the male specimen as *Pudrica christianottoi*. The placement of this species within the subfamily Aleyrodinae is on the basis of the configuration of the fore wing with a single, straight, non-forked central vein R, contrary to the pattern in Aleurodicinae where the branch Rs is present, and terminal R1 is typically short or absent. In Aleyrodinae, females are characteristic of presence of two pairs of ventral abdominal wax plates (on segments A2 or A3 and A4); this feature was not observable in female specimen from Wanninchen and Schlabendorfer See, Lower Lusatia, found in association with lignite chunks [14], due to its preservation state; male specimen with four pairs of ventral abdominal wax plates on A2–A5, clearly visible in specimen from Bitterfeld, confirming the taxonomic placement of *Pudrica christianottoi* in Aleyrodinae. Fossil resins found in Wanninchen, the area of former brown coal (lignite) mine, are the most probably redeposited, succinite was reported in association with lignite and glessite, in layers without gravel and pebbles (typical for Pleistocene strata), most probably representing Lower/Middle Miocene, Burdigalian/Langhian; Brieske Formation, Welzow Member, 2nd MFH (2. Miozänen Flözhorizontes; 2nd Miocene Seam Horizon) [42,43,44,45]. The lignite deposits that were exploited in Schlabendorf-Süd mine were formed in swamp and bog environments, under more or less marine influence, with the light micaceous sands deposited in shore face or barrier island environments; the silts representing lagoon environments and in part tidal flats as well [42,46,47]. The deposits were formed during the Middle-Miocene Climatic Optimum, and palaeobotanical data suggested various types of mixed mesophytic forests, forest- to bush moors to reed-mire vegetation, with the predominance of subtropical taxa [48,49,50].

The male specimen of *Pudrica christianottoi* was discovered in the Bitterfeld area, in the former lignite mine of Goitzsche (amber mining ceased in 1990, and subsequently the mine was flooded). This region has been recognised as a site of amber deposits for a considerable period, with records dating back to the 17th century [22,51,52]. The geological setting of the deposits of resins in the Bitterfeld area was initiated in the mid-1970s, with scientific studies of amber (succinite) inclusions commencing in the early 1980s [28,53]. To date, there have been no reports of whiteflies being present in succinite from the Bitterfeld area. In a manner analogous to the occurrence of succinite found in Lower-Middle Miocene deposits of Lower Lusatia [14], the succinite from Goitzsche mine underwent reworking and redeposition to Upper Oligocene strata, and its age is assumed to be distinctly older, with the most probable date being assigned as Middle to Upper Eocene [19,21,33,54]. At the time of (re)deposition of the resins in the amberiferous layers of Bitterfeld, the area was positioned at the northern margin of the Bohemian Massif, between 44.9 and 45.3° N [22,44,45,55,56,57]. This area has been subject to repeated changes in continental and marine-brackish facies as a result of climatic, epeirogenic and fractional tectonic shifts. During the Oligocene/Miocene transition, this region was an estuary branch of the Thierbach river system [22,58], placed in the floristic North European Province, within an area dominated by vegetation of the fossil Thierbach floral complex, which may be the source of both succinite and accessory resins [22,59,60]. The presence of the same species in geographically and stratigraphically distinct regions, albeit not very remote, may be attributable to the reworking and transportation of amber pieces during geological changes in these areas [44,45,55]. It can be plausibly hypothesised that *Pudrica christianottoi* was the species that occurred in the forests of the southern banks of the sea, and that the inclusions were spread during these events. Notwithstanding the scarce data on fossil whiteflies, the ‘taxonomic landscape’ of these insects is of considerable interest. The whiteflies inclusions from the Gulf of Gdańsk are represented by Aleyrodinae genus *Snotra* (2 spp.), and Aleurodicinae genera: *Paernis* (1 sp.), *Gregorites* (1 sp.), *Eogroehnia* (1 sp.), *Medocellodes* (1 sp.); in amber from Denmark only Aleurodicinae genus *Gregorites* (7 spp.) are present; amber from Rovno Aleurodicinae is genus *Rovnodicus* (1 sp.), while in succinite from Bitterfeld and Lower Lusatia only Aleyrodinae genus *Pudrica* (1 sp.) is known so far. Older, Lowermost Eocene inclusions in amber of Oise represent only Aleurodicinae (with monospecific genera *Clodionus*, *Isaraselis*, *Lukotekia*, *Oisedicus*). The prevalence of Aleurodicinae in the Eocene fossil records is of particular interest, as it stands in opposition to the contemporary perception of the taxonomic diversity of whiteflies. The contemporary fauna is dominated by Aleyrodinae (approx. 150 genera and over 1500 species), in contrast to the representation of Aleurodicinae comprising 21 genera and approx. 130 species. The Aleyrodinae are cosmopolitan, with the greatest diversity found in tropical and warm-temperate regions, where an estimated 63% of all whitefly species are distributed. Regarding available data, the Aleyrodinae achieved success with feeding on gymnosperms, as evidenced by their continued presence, while the Aleurodicinae do not favour these plants [9]. Representatives of both subfamilies demonstrate a marked preference for dicotyledones over monocotyledones, with the highest numbers of records pertaining to representatives of Fabales (core eudicots, rosid I clade) and Poales (monocots). The question of the potential host plant of *Pudrica christianottoi* remains unresolved; in both sites, the floristic lists of taxa encompass representatives of various groups of angiosperm and gymnosperm plants [22,28,32]. The majority of species belonging to the modern Aleurodicinae subfamily have been reported in tropical and warm-temperate Central and South America and the Caribbean. Consequently, the fossils from Europe may be indicative of the shift and ecological change reflected in the modern distribution and diversity of whiteflies.

## 5. Conclusions

Whiteflies seldom endure the process of fossilisation, thus making these discoveries of paramount importance to the fields of palaeontology and palaeoentomology. These specimens represent a significant source of information about the biodiversity and climatic conditions of the ‘amber forests’ of the Paleogene, which are believed to have been characterised by a warm and humid climate. The discovery of the *Pudrica christianottoi* specimen in Bitterfeld succinite provides further evidence in discussions about the nature, formation conditions, deposition sites and taphonomic fate of Eocene fossil resins in Central and Eastern Europe. The recent findings, accompanied by comprehensive descriptions and in-depth analyses of morphological traits and taxonomic diversity of whiteflies (utilised as a model group), in conjunction with geochemical and taphonomical data from sites containing amber deposits, and further elaborations of fossil resins and their fates within the geosphere, have the potential to enhance our comprehension of past events. These findings may serve as pivotal insights into the dynamics of rapid environmental changes in the present.

## Figures and Tables

**Table 1:** *Pudrica christianottoi*, male specimen MAIG 7290 and holotype female specimen GPIH 5083 measurements.

Measurement (in mm)	Male	Female	Measurement (in mm)	Male	Female
Body length total	1.03	1.15	Fore wing width	0.5	0.64
Head with compound eyes width	0.24	0.31	Hind wing length	0.9	1.24
Antennal segment I	0.02	0.03	Hind wing width	0.39	0.54
Antennal segment II	0.06	0.11	Profemur length	-	0.37
Antennal segment III	0.14	0.2	Protibia length	0.24	0.33
Antennal segment IV	0.03	0.1	Basiprotarsomere length	0.09	0.13
Antennal segment V	0.04	0.08	Apical protarsomere length	0.07	0.1
Antennal segment VI	0.03	0.1	Claws 1 length	0.02	-
Antennal segment VII	0.04	0.12	Mesofemur length	-	0.25
Rostrum length	0.15	-	Mesotibia length	0.27	0.37
Pronotum width	0.26	0.35	Basimesotarsomere length	0.1	0.13
Pronotum length in midline	0.02	0.03	Apical mesotarsomere length	0.07	0.1
Mesopraescutum width	0.14	0.17	Claws 2 length	0.02	-
Mesopraescutum length in midline	0.08	0.14	Metafemur length	0.25	0.3
Mesoscutum width	0.27	0.35	Metatibia length	0.37	0.5
Mesoscutum length in midline	0.03	0.05	Basimetatarsomere length	0.12	0.17
Mesoscutellum width	0.12	0.17	Apical metatarsomere length	0.08	0.13
Mesoscutellum length	0.03	0.05	Claws 3 length	0.02	-
Mesopostnotum width	0.1	-	Abdomen length including genitalia	0.62	0.6
Mesopostnotum length	0.1	-	Pygofer length	0.16	-
Metascutum width	0.24	0.13	Pygofer width	0.10	-
Metascutum length	0.04	-	Claspers length	0.11	-
Fore wing length	1.07	1.46			

## Data Availability

The original contributions presented in this study are included in the article. Further inquiries can be directed to the corresponding author.
